# Women and cities. The conquest of urban space

**DOI:** 10.3389/fsoc.2023.1125439

**Published:** 2023-05-25

**Authors:** Letizia Carrera, Marina Castellaneta

**Affiliations:** University of Bari Aldo Moro, Bari, Italy

**Keywords:** women, right to the city, urban space, international right, Agenda 2030 UN

## Abstract

In the city of the Nineteenth-century, transformed by the values of the French revolution and by the modernity, women did not have yet full citizenship. The public space was still strongly a male space and women, still with a weak public subjectivity, remained the object of the male gaze. Women have begun a process of conquering urban space by claiming their right to the city, through their physical presence in the city itself. Through physical space, women have claimed their full symbolic citizenship. The project of an inclusive city takes shape from the public demands of women who, as Annie Hockshild wrote, gave birth to the most important revolution of the 20th century. However it is a stalled revolution that still today requires a legislative protection for the project of the substantial equality, which is still not fully achieved. In addition to the various national legislations, international legislation also recognizes the central objective of guaranteeing women's right to full citizenship. In the second part of the article, the focus is precisely on the normative content of this legislation and in particular on the objectives of the UN Agenda 2030.

## 1. Introduction

Women experience a historic difficulty in their relationship with the city and with public spaces. This condition still current has among its causes, among those attributable to socio-urbanistic factors, the persistence of male models in the design and management of the spaces and times of the city and of the physical quality of public spaces that often do not guarantee an adequate level of perceived safety. These limits were often not overcome by urban policies developed by many cities. In fact, if these ones try to stem the difficulties of access and permanence of women both in public spaces and in the public sphere—the public space à* la* Habermas-, they often fail to impact on the structural level of change as denounced by the sociologist Francesca Zajcyk. As regards Italy, it is worth mentioning the Urban Times Plan in the city of Milan referred to in an interview where she points out the difficulties encountered in the implementation of Italian Law n.53/2000, which makes compulsories for all Italian municipalities with over 30,000 inhabitants to establish a “Times Office” and adopt a “UrbanTimes Plan.” Too often, the Milanese sociologist goes on, implementation has been limited to a few minimal interventions that have rendered the regulation practically pointless.

The awareness that material urban conditions can interfere, even heavily, with the women's capabilities amplifies the need to pay attention to urban models and the cultural and regulatory patterns that are underlying. At the same time, it recalls the importance of a feminine gaze capable of redefining the design of the city, of structured paths in which that gaze can be refined and of equally structured occasions of female presence in the places of administrative and political decisions.

This centrality of women and the goal of regenerating in a gender key cities and societies still socially organized on male models, is the object of an increasing recognition also at different regulatory territorial levels and up to the international one. A the more and more large number of European and international legal normative texts have progressively sought and increasingly achieved a binding objective for States, regions and, more recently for cities, of the adaptation of urban space to ensure the conditions for full female citizenship, and women's full use of the city's resources. Starting from underlying the necessary participation of women in the definition of public policies as a fundamental condition to ensure a full right to the city within accessible and socially sustainable urban contexts (Fritz, 2018).

## 2. The denied city

As Walter Benjamin showed in his iconic comment to Paul Klee's *Angelus Novus* (known as the IX thesis in *The Concept of History*), the evolution of modernity in the 19th century brought about an extraordinary transformation. It disrupted the traditional models of community and changed the experience of time, opening up a range of new possibilities and choices, and establishing reason and science as the main criteria for the explanation of the world. The city is where this transformation can be physically observed, like a “stage” that the bourgeoisie—the new political actor—uses metaphorically, to show its power and claim an economic, political and social role.

Yet, in this new city, grown out of the principles of the French Revolution, women have no citizenship. In fact, the bourgeois cultural revolution only marginally improved the quality of women's experience of the city. Their participation in the city public life is restricted by the conditions and the strict rules dictated by an etiquette, which is necessary to preserve the purity of high-ranking white women (Kern, 2020, p. 12).

Within this changing and change-shaping space, women are still only an object for man's eye. They are denied a full subjectivity in the urban space. The women that we encounter in the public space are the maids who walk fast for their tasks, the prostitutes standing in the dark corners or in the narrow alleyways of the city, the *grisettes*, the seamstresses of the Latin quarter. Differently from the typologies of women just mentioned, middleclass women live at the margins of the city's public space. They are allowed in it only if entitled by the presence of a man and his oversight, a father, a brother or husband. Women are expected to walk through the city at a brisk pace and with their head down; they should never dare to observe the city, but should always be watched, like in a sort of urban Panopticon (Ferguson, 2020). Even theaters and cafes, the typical gathering places of the bourgeoisie, remain male-centric environments, where women are seen only as a man's ornamental accessory.

According to Nesci (2007), women become victims of a urban discrimination, since their exclusion from the gendered space of the city, where they are allowed only as graceful decorations, force them into a form of aphasia. Within the bourgeois space par excellence that is the business district, women are seen as *foreigners*, with an exception made only for female clerks or secretaries. As Nord (1995) and Elkin (2016) write, women's relationship to the city is conditioned in three ways, as they are seen as an object of desire, a body for sale and a form of public exhibition. The bright windows of the shops in the French *passages* and Paris' shops full of lights and goods will be, in women's eyes, an opportunity to grab their limited portion of public space. Despite the limits and risks coming with consumerism, they begin to earn a partial and illusory right to the urban experience of the city, although this continues to be a space for men. As Émile Zola writes in “Au bonheur de dames 1883,” this chance to take over the public space is nothing but a *poisoned gift* (Zola, 1883). Also, Adorno and Horkheimer pointed out that women's view of the city remains one of subordination, because it is filtered by the glass of the shop windows or those of their own house. Since the public space is still forbidden to them, in terms of a political space of conversation and discussion, women's participation is partial and fails to make its way into the everyday life of the modern city.

But, the 19th century was a time full of complexities and contradictions and in the same urban spaces there were the *flâneuse*, as George Sand, Olympia de Gouges and Flora Tristan, who wander around the city, sometimes in male clothes, to be able to enjoy it all on their own. That emerging in the space of the new modern city is a new kind of woman.

Even if it is not a concept fully shared even within the same feminist reflection. The existence of *flâneuse*, an example of this new female way to live and experience the city*, indeed*, is not without disagreements. For example, Wolff (2006) writes about the recognition of the role of women in public spaces has been belated, stressing the female status of object of men's gaze, and Pollock (1992) argues the absence of women precisely starting from the gaze of the *flâneur* who observes the city and the women was a form of possession, of appropriation through sight, and argues that there could not have been a female correspondent of that look. Duffy (1994), while not completely excluding the presence of this figure, believes that the essential conditions of anonymity in which the *flâneur* moves invisible in the crowd, were certainly denied to women, always very much visible (Hallberg, 2002; Nuvolati, 2006, 2013, 2014).

Although these opposing positions cannot be neglected, on the contrary there is a broad agreement on the existence of the *flâneuse* as a symbol of a new and different presence of women who claim a leading role in the urban space (Wilson, 1992; Nesci, 2007; Elkin, 2016; Carrera, 2022b). Already in the 18th century, if on one hand, gender models based on the exclusion of women from political participation continued to prevail, on the other hand, there were movements, like that of the suffragettes, that advocate for women's rights by physically taking the streets of the cities to reclaim their public space. Here, citizenship is all but an established fact, especially for women. It is rather a fluid, dynamic process involving negotiation and struggle (Lister, 2003; Tastsoglou and Dobrowolsky, 2006). As Lefebvre (1968) will write a century later, a real revolution is always an urban revolution, that is, it can only take shape in the city's public space. Hannah Arendt argues that political action always needs a “space of materialization,” and the city does provide this site for women, although in an uncertain way.

While important steps are taken in the 19th century to encourage women's leading role in the urban space, it is in the 20th century that women's agency comes fully to the fore. As Arlie Hockshild points out, the female revolution generated changes in the everyday life penetrating deeply both the normative and cultural level, the everyday life as well as the local and the international level. As Hockshild pointed out at the end of the 80s, this is, however, a “stalled revolution,” with alternate phases of decline and progression, objects of resistance and a patchy pattern of accomplishments. Forty years later, Hockshild's remarks do not sound so unrealistic and anachronistic, as the continuation of traditional gender representations is still a main concern of large part of the literature on patriarchy (cf. among others, Garavaso and Vassallo, 2000; Allouchi, 2020; De Vita, 2020).

Still now, the city envisaged by Habermas as a public, political space is something that women cannot take for granted.

## 3. The sexed urban space

As Cacciari (2009) writes, the city is a place where the man acts as a creator and builds it to his own image. Hence, social injustices, polarizations, contradictions and limits are embedded in the social, economic and cultural structures that give shape to the city, indelibly imprinted in its spatial arrangement. The city looks like the unequal space pictured by Secchi (2016) or, as Balbo writes (1987), it is fragmented and divided into “microstates,” within which the quality of life depends on the individual's financial resources (Harvey, 2008, 2012, 2014). In this post-metropolitan scenario, which is characterized by a lack of community spaces and ties, the city is no more an authentic place, but turns into a simple territory for cohabitation (Cacciari, 2009).

From a gender perspective, this urban and cultural geography rests on the awareness that those who observe, design and draw the lines of the city are not unbiased observers (Williams, 2016). The sexual space confirms, on a physical level, the traditional, symbolic and normative gender order which operates in social contexts (Monk and Hanson, 1982; Darke, 1996; Hooks, 2000). Thus, Darke's (1996) remarks about the non-neutral character of the cities are still relevant today. She argues that every settlement is an inscription of the patriarchy in stone, bricks, glass and cement. After the cities are built, they continue to shape and influence social relationships, power, inequality. They contribute to settle some disputes by making things normal and right, while other issues are misplaced or even settled in a wrong way. Space contributes to building the same concept of gender that varies in time and space (Cream, 1995). Urban space is part of a series of social norms, mandatory relationships and narratives that lead to the naturalization of certain worldviews and of oneself (Borghi and Rondinone, 2009, p. 63). This analysis draws on an account of the suburbs—the byproduct of rationalist urban planning—like that of Wekerle (1984), who interpreted them as the spatial reconfirmation of the role reserved by men to mothers and wives (Kern, 2010, 2020). This had also been pointed out before by Jacobs (1961), as she argued that the city, not the suburbs, should have been considered as women' space.

In the 19th century, the city and the right to its public spaces return to be the scenario and target of strong claims. As Predelli (2008) writes: “A current trend in feminist studies of ‘citizenship' is the opening up of the term from a narrow political-legal definition to a broader and more inclusive cultural-social definition, and, subsequently, attempts to analyze the extent to which women (…) exercise citizenship in this broader sense (…). The classic view of citizenship as delineating legal and political rights and duties has been challenged on several fronts, including its limitation of citizenship to the public sphere and its narrow view of citizenship as ‘status'.”

The right to the city implies much more than the simple access to the material resources that the city offers: it is a right to change, reinvent and re-design the city. Inevitably, this depends on the exercise of a collective power on the process of urbanization. As Harvey (2016) points out, the right to the city entails a claim to decision-making power over the urbanization processes and the city's re-development. Women, however, started to discover and use this urban and political agency less than two centuries ago, both in a symbolic and in a physical way that changes and re-signifies the political connotations of public space and places.

Drawing on Roman Jakobson's analysis of metonymy, De Vita points to the responsibility of the bodies, writing that the bodies are responsible “*for their full being, and redesign the spaces of the city through their sense and dissent, between physical and symbolic boundaries*” (De Vita, 2020, p. 47, the translation is mine).

Such remarks provide a better ground to understand the idea of re-shaping, re-subjectivizing, and re-signifying human spaces as a metonymic re-affirmation of dissent and of the claims to determine the ethical and political relations through spatialization. The focus, Caravero (2019) notes, is on the materiality that defines a full experience of the space.

Women's contribution to re-define public spaces in terms of the ties that they make possible can help fragile territories not to fall apart, and enable connections that, within the patriarchal tradition, would not be permitted (De Vita, 2013).

The so-called *third spaces* (Carrera, 2022a) are places of mutual encounter, recognition and relation. They enhance the subjective and physical experience of space and allow room for negotiation between institutions and social reality, which can also be a way toward social empowerment (Alga and Cima, 2020, p. VIII). As they lie between public and private, the third spaces are in the position to influence both these levels. Their cultural identity is shaped through a plurality of small, almost interstitial places scattered throughout the city, which provide opportunities to activate strategies for inclusion.

Originally, it was Bhabha (1990, 1994) to point to the hybridization of differences as a process to locate in a ≪third space≫, that is, an area of continuous negotiation of meanings and representations, through which given hierarchies and consolidated conventions could be unsettled, and new opportunities arise. This also means that the “third space” between the actual city and the regulated spaces, is the place of uncertainty, like a gap through which change can become real and processes of mutual recognition between differences, including gender ones, can take place by rethinking and redefining urban spaces.

Soja (1996, 2000) re-conceptualizes the notion of *third space* pointing specifically to the aspect of symbolic representation closely related to it, in particular, the radical changes and creative responses to changes that are constructed and deconstructed within the new liminal areas of the urban space. Soja can, thus, be mentioned next to Habermas and Lefebvre, as scholars that thought of a third space in terms of encounter between different people, be a short interaction or a more stable association to generate ideas and practices that can have political implications. To use Habermas' words (1984), the city, and especially its *third spaces*, is where the memory of a *public sphere* is created (Jedlowski, 2016).

## 4. Women's gaze and the other city

The creative potential of physical and immaterial spaces can be a starting point for thinking differently about the city. The *women's gaze* has the potential to change what we “take for granted,” and undermine and destabilize what we consider “natural” (Butler, 1990). As Tremblay (2000) argues, the third stage of reflection on women's political activity suggests that women have an impact on politics; it fails, however, to address the exact nature of this impact. The new notion of *flânuserie*, with its political potential, is an attempt to explore at least part of this problem. For example, Lefebvre's conception of *other city* aims to design an urban habitat able to fully restore subjectivity and citizenship through an *eccentric logic*, which requires bringing urban and symbolic boundaries at the heart of public debate (Alga and Cima, 2020). The idea of an *oblique gaze* (Carrera, 2015, 2021, 2022b), that diversity can awaken, entails a movement between different viewpoints or perspectives (cfr. Hartsock, 1989, 1997). These views are necessary to produce “a powerful, critical discourse that is epistemically effective” (Longino, 2002, p. 131). Women's gaze shows exactly the breaking through of the difference and finds a full expression when heterotopic groups realize the possibility of collective action to create something radically different (Harvey, 2012, 2014).

This role can only be played by those women who are capable of critical and reflexive thinking in relation to different perspectives, what Mills (1959) called *sociological imagination*; they recognize the city as the place of limitations as much as of possibilities, and not only as the materialization of the dominant normative models, but also of *other* possibilities beyond the given reality.

In other words, modern *flâneuses* make of their urban experience an opportunity to disclose its hidden ideologies and re-design existing social patterns. Two centuries after the first appearance of *flâneuses* in the streets of Paris and of other European cities (Nesci, 2007; Elkin, 2016; Carrera, 2021, 2022b), and despite the suffragettes and other feminist movements of the second half of the twentieth century, women are still in the grip of a difficult citizenship within the urban space. They continue to be at the margins, in a city that is not the same as the men's one. Their gaze still speaks about difference and provides that essential perspective to re-think and re-design the urban system. What Simmel wrote more than a century ago remains relevant today: ≪women, as such, not only have a different mixture of equality and inequality with historical objects, they have not only the opportunity to see more things than men; (...) they also know how to see the same things differently (...) in the same way also the historical world, mediated by the psychological interpretation of women, could acquire a different partial and overall aspect≫ (Simmel, 1890).

Women's gaze and presence in the city is a revolutionary act in itself, for it disrupts the domestic condition in which they have been confined. Women's “eyes on the road,” to use Jacobs's (1961), have the democratic potential not only to widen the number of people called to make decisions about the city, but to change the assumptions on which such decisions are made.

It is important, here, to highlight the radical difference of Lefevre's concept of heterotopy from that of Michel Foucault. The former points to liminal social spaces that are rich in possibilities leading to revolutionary trajectories. However, the vision of “something that can be different” does not necessarily arise from a conscious project, but it simply emerges from what people do, feel, perceive and are able to express in making sense of their everyday life. As liminal subjectivities think differently and see things in a different way from others, women's *thoughtful gaze* enables readings of the city that include an understanding of both the interstitial and the systemic. This means that the gazes and practices of different subjectivities transform the physical city from a single into a plural phenomenon. In other words, the urban “mindscape” opens up and is enriched by new representations.

Women's different experience of time and space makes their relationship with urban spaces particularly complex (Balbo, 1987; Gordon, 1989; Pain, 1991, 2001; Valentine, 1998). Through their networks, they create their own geography and city map. Still now, women's experience of the city is different from men's one and more challenging than that. It is, at once, more intense, because of women's many duties of care, and more limited, because its boundaries are defined by the geography of fear (Carrera, 2015; Kern, 2020).

The difference embedded in women's gaze is, therefore, a useful guiding principle for a participatory process of urban re-design and regeneration. As noted, to achieve the goal of an inclusive city, women need to understand their experience of the city as an opportunity for reflective and political practice.

Female thinking reinforces the idea of the city as an extended “construction site,” an always open and problematic space, where groups with different interests confront each other and often clash (Harvey, 2014). In this dynamic scenario, the thoughtful gaze of women could gain an unprecedented centrality, by making visible again what in the urban normality has become invisible for the hasty citizens. The modalities of female thinking are such that women can be considered as part of the city, but also, in some way, disconnected from it. They combine their attention to the small details and the particular circumstances of the urban experience with the ability to re-connect those fragments to a more general picture, reassembling different temporal and spatial elements into a coherent vision of the city. This thinking also offers the chance to envisage the opportunity to disrupt existing patterns and routines (Bourdin and Masboungi, 2004) of the urban regeneration processes.

To ensure that women are able to express this potentiality for change, it is essential to include new spaces and opportunities for women's political participation within the current legislation. It goes without saying that women's full citizenship should not only be defined in terms of a balancing of rights between men and women, but also in relation to women's affiliation to minority or non-minority groups, their ethnicity, origin and urban or rural residence (Yuval-Davis, 1997). When reflecting on the public space that women have the right to claim, it is essential to include an intersectional element. This is the key point, in summary, to go beyond the political rhetoric, and make sure that women's participation in the process of policymaking, first and foremost in relation to the urban space, is intended as an opportunity as much as a need for cities to become fully inclusive. The female gaze, as well as that of other differences, can help to pursue the goal of making the city a *welcoming space* starting from the questioning of the heteronormative model.

Starting from the recognition of the role that urban space has, both materially and symbolically, toward the aim of a full women social citizenship and of a subversion of traditional gender patterns, international law has given increasing prominence to the role of cities in the pursuit of objectives of protecting diversity and guarantee full gender equality in the quality of social and political life.

## 5. International acts protecting women as a tool to ensure women's social and political participation

If, at first glance, the role of cities might appear to be of no interest for international law, recently, this topic has been given the right emphasis in the international community^1^ as a consequence of the action plan issued by the General Assembly of the United Nation on the 21st October 2015 (A/RES/70), also known as Agenda 2030, which consists of 17 Sustainable Developments Goals (SDGs), one of which—No. 11—focuses, specifically, on cities.^2^ This is something totally new from the previous program, the 2000 Millennium Development Goals, which did not make any mention of the local level, while both global and local action are explicitly mentioned in the Agenda 2030 as key aspects for citizens' engagement in the pursuit of the program's several targets.^3^ Also, women's access to public urban spaces is mentioned as a specific requirement of the SDG No.5 on gender equality. In brief, Agenda 2030 highlights that regulatory action in relation to cities and to the role of local authorities is an essential aspect of sustainable development. It is precisely the circumstance that international law has not dealt with the relationship between cities and women that leads us to focus our attention on the Agenda 2030 and on a central aspect, i.e., security in cities as a means of ensuring women's participation in society and the full realization of human rights.

The centrality of cities for an effective implementation of some international principles and values is, thus, recognized in its close connection to people's safeguarding and human rights' protection, including women's right to participate actively in the democratic life.^4^ This means, for example, that the benchmark alone concerning a minimum standard of gender balance within candidate lists for local election is not an effective measure. To ensure a full and effective engagement, women should be allowed to play a more central role in the urban planning, and be involved in the prior phases of local councils' decision-making process. This is also suggested, for example, in the Recommendation of the Target No. 11.7 of Agenda 2030 to provide “access to safe, inclusive and accessible, green, and public spaces, particularly for women and children, older persons and persons with disabilities.” (SDG No. 11). These new measures are in line with a long-lasting concern of international law with the protection of women' rights, with a particular emphasis on the importance of State regulatory action that takes into account the status of women as a vulnerable group influenced by stereotypes deeply rooted in society.

Within the context of international legal tools that can be used to improve the urban environment and make it more supportive of women's active participation, it is worth mentioning not only the Convention on the Political Rights of Women (New York, March 31, 1953), but also the Convention on the Elimination of All Forms of Discrimination Against Women, a treaty commonly known as CEDAW, issued by the General Assembly of the United Nations on the 18th of December 1979, 4 years after the First World Conference on Women. The Treaty is the first comprehensive international bill of women's rights that aims to address gender inequality and discrimination, not just by prohibiting discrimination and violence against women, but also by introducing positive measures to be taken at national and local level. The Treaty states that “Countries must take action to promote equality and end discrimination against women and girls, by establishing laws and policies to protect them from discrimination” (Article 2),^5^ and compels governments to take “all appropriate measures to eliminate discrimination against women in the political and public life of the country” (Article 7). In addition to ensure that women's rights are protected by State law, such measures are intended to promote more concrete action at local level. The Treaty has, thus, provided a general framework to bring the global into the local in order to empower women's role in the city. For example, the city of San Francisco, in the United States, adopted an order that reflected CEDAW's principles, although the USA did not adhere to the Treaty.^6^ This clearly shows the importance of the local level in the implementation of international law, as already the 1993 World Conference on Human Rights had pointed out.

Another achievement of the 1979 Convention is the setting up of a committee—“the United Nations Committee on the Elimination of All Forms of Discrimination against Women”—to permanently monitor the countries' progress in the implementation of the goals and identify critical issues and best practices at national level.

Despite this regulatory effort at international and regional level, which includes similar initiatives in the European and South-American area—such as the European Council Convention on preventing and fighting violence against women and domestic abuse (Istanbul, May 11th, 2011) and the Inter-American Convention on the Prevention, Punishment and Eradication of Violence against Women adopted by the Organization of American States (Belem do Para, June 9th, 1994)—women's right to participate in the public life is undermined by a surge in acts of violence against women worldwide.

This shows that an effective protection of women's rights and the implementation of fundamental freedoms require appropriate regulatory action in relation to safety in the urban space.^7^ This issue has received far less attention, although it is closely linked to women's individual and collective fulfillment. Future legislation should aim, therefore, to address the limits of the international regulatory system, whose binding power can only be exercised against the national level. The way toward an effective involvement of local communities was originally laid out in a regional context, with the adoption of the European Charter for the Safeguarding of Human Rights in the City,^8^ at the 2001 Second Conference for European Cities held in Saint Denis.^9^ The UN's treaty has further highlighted that sustainable development depends on global as well as a local action, and, most importantly, on that of the citizens. This relationship will be explored in more detail in the following section.

## 6. The relationship between women's rights and sustainable cities: The SDGs and the Agenda 2030

International treaties continue to play a pivotal role in the improvement of women's status and socio-political participation. However, the more coherent framework introduced by the Agenda 2030, has the potential to enable structural and wide-ranging changes to promote the affirmation of rights within the concrete context of people's lives. The adoption of the SDGs has been particularly effective in raising the attention on the role of cities in the implementation of the rights guaranteed by international law, precisely because the SDGs require specific action to be taken at local level. Several other initiatives show a revived interest in this field, like the new research group on cities and international law established by the International Law Association^10^ and the New Urban Agenda launched during the 2016 World Habitat III Conference in Quito (Ecuador). The latter, in particular, recognized the cross-cutting nature of urban issues and the interrelatedness between these and other Sustainable Development Goals.^11^ In particular, this document calls for cities “to be secure, positive, respectful, and safe places for all people to live and work without fear of violence or intimidation recognizing that women and girls are disproportionately affected by violence in cities and all human settlements.”^12^

The 2030 Agenda has further specified that local authorities must be held to account by reporting on the progress of the Agenda's implementation (see § 89), thus driving and facilitating a multilevel participation process including international, national and local levels. By defining cities as hubs “for ideas, commerce, culture, science, productivity, social, human and economic development,” the Agenda also enables a close connection between the quality of city life, and urban mobility, which is crucial to make cities and human settlements inclusive, safe, resilient, and sustainable (SDG No. 11, “Sustainable Cities and Communities”).

As mentioned before, gender equality is a core element of the Agenda, explicitly addressed in the SDG no. 5, which requires States “[to]Achieve gender equality and empower all women and girls.” A central assumption of the Agenda 2030 is that gender inequality represents a major obstacle to sustainable development and that this includes the elimination of all forms of violence against women (specifically indicated in target No. 5.2). This implies that, along with measures to eliminate the violence that triggers discrimination, it is also important to combine the achievement of fundamental human rights, i.e., gender equality, with collective action to abolish discriminatory laws that are causing women's underrepresentation at all levels.

Despite the growing awareness on the centrality of women's empowerment for the achievement of sustainable development, with particular emphasis on cities, recent data reveal a very challenging situation for women. Presenting the 2020 annual report on the state of implementation of the SDGs, the Secretary General of the United Nations, António Guterres, has warned that the achievement of the Goals by 2030 has become an unrealistic target, since violence against women has increased up to 30%, and the implementation of the SDGs, probably because of the COVID-19 pandemic, has slowed down. While women's representation in leadership roles is increasing, women themselves “are facing new barriers and new threats, ranging from a shadow pandemic of violence to additional burdens of unpaid care work.” The interrelatedness between the different SDGs, and in particular between the SDG on women and that on cities, might easily explain the slowdown in the progress toward SDG No. 5. The report on SDGs' indicators shows indeed that the implementation of SDG No. 11^13^ was significantly affected by the lockdown and the “closure” of open public spaces, meaning that women's participation in city life was also reduced.

In the following section, the relationship between women's empowerment and cities' sustainable development (respectively, SDG No. 5 and SDG No. 11) will be explored in more detail in relation to the evaluation of the Agenda 2030's implementation and its monitoring systems.

## 7. The monitoring system and the sharing of best practices

In addition to ensuring greater effectiveness in promoting change that is supportive of women's participation in the urban context, the approach of the SDGs is particularly innovative for its monitoring system, which allows to identify effective measures and best practices adopted by the public as well as the private sector, both at state and local level. This system enables a continuous interaction among the stakeholders that contributes to the improvement of society as a whole. Let us see more in detail what this system involves.

Firstly, the United Nations have engaged with the High-Level Political Forum on Sustainable Development (HLPF), which was established at the 2012 United Nations Conference on Sustainable Development (Rio +20), also known as “The Future We Want.” Moreover, with a resolution adopted on July the 9th, 2013,^14^ the General Assembly has provided guidance and recommendations on the format and organizational aspects of the SDGs' implementation and review process. It has also ruled that the Forum's meetings “shall be convened under the auspices of the General Assembly and of the Economic and Social Council” at the level of Heads of State and Government,^15^ although the participation of relevant stakeholders is also expected. The eight-days long annual meetings of the Forum are currently considered of such importance that the General Assembly established, with Resolution 70/299 of July 18, 2016, that the HLPF will be also responsible for the follow-up of the SDGs implementation,^16^ pursuant to paragraph no. 84 of the Agenda.

To stress the importance of the national level, a system of “Voluntary National Review” (VNR) was set up during the July 2018 meeting of the HLPF as a tool for subnational levels of government to take on SDGs.

Within this system, States are not only required to show progress in the SDGs follow-up in their annual report through an effective assessment scheme, but also to share their practices through an online review platform.^17^ As part of this process, the Italian government presented a National Sustainable Development Strategy (NSDS) in its 2017 report and the second Voluntary National Reviews in 2022. The Strategy illustrates a range of reforms that this country plans to enact by 2030 to grant women equal rights in the access to economic resources, as well as in the ownerships and control over land and other forms of property, financial services, inheritance and natural resources. These measures are intended to align the national laws with the SDG no. 11, which requires States “[to] provide universal access to safe, inclusive and accessible, green and public spaces, in particular for women and children…” (p. 50). Given the frequency of physical and sexual abuses on women across Italy, it is essential that National Strategic Goals include measures to “prevent violence against women…” as a necessary step to promote a non-violent and inclusive society (SDG No. 16, Peace).

A concerning picture regarding women's safety in Italy emerges from the National Institute of Statistics (ISTAT), the Italian agency that coordinates the national indicators for the measurement of sustainable development and monitors its objectives, as required by the United Nations Statistics Division (UNSD). ISTAT's last report on target No. 5, released on August 9th, 2021, shows that 20.2% of women between 16 and 70 years-old experience violence in an open space, compared to 18.5% in their own home,^18^ a figure that again points to the importance of a joint implementation of different SDGs. Further evidence is provided in Table 5.1, which illustrates relevant data in relation to Goal No. 5.2.1. The table shows that the number of women that reported themselves as victims of violence to the public utility number against violence and stalking has been steadily increasing in the last 10 years as well as in comparison to last year.

## 8. Achieving the global goals in renewed, sustainable cities: Some practical examples

As mentioned above, the implementation of the SDGs must be conceived as both a top-down and a bottom-up, comprehensive process. This means that local authorities (countries, cities, and so on) should be encouraged to participate more actively in the broader system of the Voluntary National Review (VNR). Action at local level is useful not only because it allows for more targeted interventions, but also because it creates opportunities for cities of different sizes to share successful practices in relation to the achievement of the so-called “human rights cities,” i.e., cities that apply international law and, in particular, human rights and SDGs.^19^ The recently introduced sub-national review system (Voluntary Local Reviews)^20^ works precisely in the direction of promoting local authorities' participation in the process of SDGs' implementation.

Interestingly, no Italian city has joined the Voluntary Local Reviews initiative so far, despite Italy's participation in the High-level Political Forum, whereas many big and small Finnish, German, even Brazilian cities submit regularly a report to the Department of Economic and Social Affairs of the United Nations, in accordance with the Global Guiding Elements for Voluntary Local Reviews and other available guidance in relation to SDGs implementation.^21^

The lack of engagement from Italian cities is extremely concerning, given the centrality of local and regional government in supporting cities and regions “to foster SDG localization and demonstrate local governments' capacity and commitments,” as the High-Level Political Forum has highlighted several times in the VNRs 2020.^22^

Beyond the Italian case, it might be useful, here, to illustrate how other cities plan to achieve Goal No. 5 in connection with Goal No. 11, and what are the elements they see as key in this respect. So, it seems useful to dwell, considering the impossibility of analyzing every single city that participated in the VLR, on some cities in South America that have very high rates of violence against women and cities like Helsinki that have long been active in security.

Evidence from the submitted reports shows the need to focus on safety in public urban spaces, for example, by improving the lighting and increasing the number of bus stops in the streets.

Mexico City, a town with nine million people, 52.2% of which are women and 47.8% men, with serious problems of violence and women's marginalization, has started to apply the VNR system, and can now offer an example of participation to other Mexican cities. Its report includes a specific focus on the measures implemented and planned by the local government to prevent violence against women and ensure their rights are protected (*Derechos des la mujeres*). As part of the SDGs' implementation, the local government is also operating a system, the “Sistema Universal de Cuidados,” which guarantees universal assistance to job seekers, and particularly encourages women to seek employment, supporting them if they need to work outside their home. In its 2021 report, the City of Buenos Aires, has given a special priority to the implementation of SDG No. 5, and has explicitly mentioned that it will promote “cross-cutting initiatives so that men and women have the same opportunities to develop.” Many of the initiatives mentioned in the city's report aim at encouraging women's participation, linking action in this area to measures for mobility improvement, as travel safety helps accessing services and participating in public life. In the specific section on “Gender Equality,” the city of Buenos Aires' report states that “The Comprehensive Strategy for Gender Equality (launched in 2018) seeks that all women of the city can transit and enjoy public spaces without violence.” In other words, Buenos Aires' local government believes that women' safety is essential for them to lead economic development and play a role in decision-making processes. It is worth noting that, following the publication of Agenda 2030, a statistical system has been established to collect specific data on the condition of women, known as Gender Indicators System (SIGBA), which includes 92 indicators “classified under the three autonomies of women: physical, economic, and in decision-making.”

The city of Helsinki offers a prime example of a city that has already implemented many of the SDGs. In its report entitled “From Agenda to Action: Implementation of the UN Sustainable Development Goals in Helsinki (2021),” the local government has stressed that “gender equality is a principle permeating all activities of the city” and every action and project launched in the city takes into account the impact on women. The city of Bonn, in Germany, has also presented a report, and takes part in regular VLR. The report, “Agenda 2030 on the local level: implementation of the sustainable development goals in Bonn,” states that the city has established “The Competence Center for Women & Work” to increase the percentage of women in senior roles, including in the town's administration, and to support women in pursuing a career. Furthermore, as stated in the report “Barcelona: Sustainable Future,” the city of Barcelona has enacted 16 “Municipal strategies and plans for the localization of SDG 5,” specifying that women's access to safe, inclusive, and accessible public spaces is a key point to achieve this goal.

Just these few examples show that a model for the implementation of women's rights recognized at international level exists. The Italian cities should look at those examples to move toward more concrete plans for the achievement of SDGs, ultimately, by starting to participate in the VLR system.

## Author contributions

LC wrote introduction and paragraphs 2, 3, and 4. MC wrote paragraphs 5, 6, 7, and 8.

## Footnotes

^1^On the status and the role of cities and transnational city networks in International Law, see Francioni (2020) and Nesi (2020).

^2^See Aust (2020), Haddad (2021), Schroeder (2021), and Swiney (2021).

^3^Un Secretary General António Guterres, report on “Decade of Action,” adopted on 7 January 2020.

^4^Singh (2005) and Oomen and Baumgärtel (2018a).

^5^See Sisson and Sanders (2021).

^6^See the initiative “Cities for CEDAW,” launched in 2014, joined by, among the others, big cities like Los Angeles, New York: see at http://citiesforcedaw.org. On the topic, see Lozner (2008), Campbell (2018), and Saccà (2018).

^7^See *Safe Cities and Safe Public Spaces*, published by *UN Women Global Flagship Programme*, available on the website https://unwomen.org.

^8^ https://uclg-cisdp.org/sites/default/files/documents/files/2021-06/CISDP%20Carta%20Europea%20Sencera_baixa_3.pdf

^9^https://europe.ohchr.org/EN/NewsEvents/Pages/DisplayNews.aspx?NewsID=2579&LangID=. See also the website https://humanrightscities.net/human-rights-cities/, as well as the network composed of 200 cities from 38 countries (15 Italian ones are present) https://eurocities.eu/.

^10^See “The Role of Cities in International Law,” at https://ila-hq.org.

^11^Un Department of Economic and Social Affairs, in https://sdgs.un.org.

^12^See the document of the UN Women, “Gender Equality and the New Urban Agenda,” at https://unwomen.org.

^13^See the chapter “The need for data innovations in time of COVID-19” at page 4 of the report, on the website unstats.un.org. See also the Report “Progress toward the Sustainable Developments Goals” (E/2021/58, 30 April 2021).

^14^A/Res/67/290.

^15^The HLPF is chaired by the President of the Economic and Social Council.

^16^See also Resolution 75/2090 adopted on 28 June 2021 and before, Resolution 70/299 of 29 July 2016.

^17^See https://sustainabledevelopment.un.org.vnrs.

^18^These data, however, refer back to 2014 and have not been updated. For more information, see the documents at http://www.istat.it.

^19^See Oomen and Baumgärtel (2018b). See also Oomen (2019).

^20^https://sdgs.un.org/topics/voluntary-local-reviews.

^21^For example, see The European Handbook for SDG Voluntary Local Review published by the European Commission's Joint Research Center and available at https://publications.jrc.ec.europa.eu.

^22^Concept Note, High-Level Political Forum, 2020, https://sustainabledevelopment.un.org.

## References

[B1] Alga M. L. Cima R. (2020). Allargare il Cerchio. Pratiche per una Comune Umanità. Bari: Progedit.

[B2] Allouchi N. (2020). “Dar Rayhana: pratiques quotidiennes de femmes,” in Allargare il cerchio. Pratiche per una Comune Umanità, eds M. L. Alga, and R. Cima (Bari: Progedit), 163.

[B3] Aust H. (2020). Cities as international legal authorities. J. Comp. Urban Law Policy 84, 2.

[B4] Balbo L. (1987). Time to Care. Politiche del Tempo e Diritti Quotidiani. Milano: Franco Angeli.

[B5] Bhabha H. K. (1990). Nation and Narration. New York, NY: Routledge.

[B6] Bhabha H. K. (1994). The Location of Culture. New York, NY: Routledge.

[B7] Borghi R. Rondinone A. (2009). Geografie di Genere. Milano: Unicopli.

[B8] Bourdin A. Masboungi M. (2004). Un Urbanisme des Modes de vie. Paris: Le Moniteur Editions.

[B9] Butler J. (1990). Gender Trouble: Feminism and the Subversion of Identity. New York, NY: Routledge.

[B10] Cacciari M. (2009). La Città. Milano: Meltemi.

[B11] Campbell M. (2018). Women, Poverty, Equality: The Role of CEDAW. Oxford.

[B12] Caravero A. (2019). Democrazia Sorgiva. Milano: Raffaello Cortina Editore.

[B13] Carrera L. (2015). *Vedere la Città*. Gli Sguardi del Camminare. Milano: FrancoAngeli.

[B14] Carrera L. (2021). “*La flâneuse. Lo Sguardo Obliquo Nella Città*,” in Fuori Luogo. Rivista di Sociologia del territorio, Turismo e Tecnologia, Anno V - Volume 10, 15–28.

[B15] Carrera L. (2022a). Designing inclusive urban places. Ital. Sociol. Rev. 12, 141–158. 10.13136/isr.v12i1.522

[B16] Carrera L. (2022b). La Flâneuse. Sguardi ed Esperienze al Femminile. Milano: Franco Angeli.

[B17] Cream J. (1995). “Resolving riddles. The sexed body,” in Mapping, eds D. Bell, and G. Valentine (London: Routledge).

[B18] Darke J. (1996). The man-Shaped City, in Chris Booth, Jane Darke, Sue Yeandle (a cura di), Changing Places: Women's Lives in the City. London: Sage.

[B19] De Vita A. (2020). “I territori delle donne. Gli spazi dei legami,” in Allargare il cerchio. Pratiche per una comune umanità, eds M. L. Alga and R. Cima. p. 42–52.

[B20] De Vita R. (2013). “Donne in percorsi non tradizionali. Tra nuove opportunità e vecchi limiti,” in Lavoro e ricerca sociologica. Un confronto tra giovani ricercatori italiani, eds M. La Rosa and U. Pallareti (Milano: Franco Angeli).

[B21] Duffy E. (1994). Disappearing Dublin: Ulysses, Postcoloniality, and the Politics of Space. Attridge and Howes.

[B22] Elkin L. (2016). Flâneuse. Women walk the City in Paris, New York, Tokyo, Venice and London. London: Vintage.

[B23] Ferguson S. (2020). Women and Work: Feminism, Labour and Social Production. London: Pluto Press. 10.2307/j.ctvs09qm0

[B24] Francioni F. (2020). Cities and countryside: an international perspective. Ital. Yearb. Int. Law Online 30, 155–165. 10.1163/22116133-03001009

[B25] Fritz J. M. (2018). “Cities for CEDAW: notes on the road to effective intervention,” in Democracy, Power and Territories, ed F. Saccà (Milano: FrancoAngeli), 1.

[B26] Garavaso P. Vassallo N. (2000). Filosofia Delle Donne. Bari-Roma: Laterza.

[B27] Gordon M. T. (1989). The Female Fear. New York, NY: Free Press.

[B28] Haddad H. (2021). When Global Becomes Municipal: Cities Internalizing International Human Rights Law. Available online at: https://ssrn.com (accessed July 2022).

[B29] Hallberg U. (2002), Lo Sguardo del Flâneur. Milano: Iperborea.

[B30] Hartsock N. (1989). “Postmodernism and political change: issues for feminist theory,” in Cultural Critique, eds N. Hartsock, D. Przybylowicz, and P. McCallum (Cary, NC: Oxford University Press), 15–33. 10.2307/1354291

[B31] Hartsock N. (1997). Comment on Hekman's “Truth and method: feminist standpoint theory revisited”: truth or justice? (journal)*Signs*. 22, 367–374. 10.1086/495161

[B32] Harvey D. (2008). The right to the city. New Left Rev. 53, 23–40.

[B33] Harvey D. (2012). Rebel Cities: From the Right to the City to the Urban Revolution. London and New York, NY: Verso.

[B34] Harvey D. (2014). Seventeen *Contradictions and the End of Capitalism*. Oxford: Oxford University Press.

[B35] Harvey D. (2016). Il capitalismo contro il diritto alla città. Neoliberalismo, urbanizzazione, resistenze. Bologna: Ombre corte.

[B36] Hooks B. (2000). Feminist Theory: From Margin to Center. Cambridge: South End Press.

[B37] Jacobs J. (1961), The Death and Life of Great American Cities. New York: Random House.

[B38] Jedlowski P. (2016). Intenzioni di Memoria. Sfera Pubblica e Memoria Autocritica. Milano: Mimesis.

[B39] Kern L. (2010). Selling the “Scary City”: gendering freedom, fear and condominium development in Neoliberal City. Soc. Cult. Geogr. 11, 209–230. 10.1080/14649361003637174

[B40] Kern L. (2020). Feminist City: Claiming Space in a Man-made World. London and New York: Verso.

[B41] Lefebvre H. (1968). Le droit à la Ville. Paris: Éditions Anthropos.

[B42] Lister R. (2003) Citizenship: Feminist Perspectives, 2nd ed. New York, NY: Palgrave Macmillan. 10.1007/978-0-230-80253-7.

[B43] Longino H. (2002). The Fate of Knowledge. New York, NY: Paperback. 10.1515/9780691187013

[B44] Lozner S. L. (2008). Diffusion of local regulatory innovations: the San Francisco CEDAW ordinance and the New York City human rights initiative. Columbia Law Rev. 104, 768–800. 10.2307/4099330

[B45] Mills C. (1959). The Sociological Imagination. New York, NY: Oxford University Press 10.2307/1891592

[B46] Monk J. Hanson S. (1982). On not excluding half of the human in human geography. Prof. Geogr. 34, 11–23. 10.1111/j.0033-0124.1982.00011.x

[B47] Nesci C. (2007). Le Flâneur et les Flâneuses. Les Femmes et la Ville à L'epoque Romantique. Ellug, Grenoble. 10.4000/books.ugaeditions.4220

[B48] Nesi G. (2020). The shifting status of cities in international law? A review, several questions and a straight answer. Ital. Yearb. Int. Law 30, 17. 10.1163/22116133-03001003

[B49] Nord E. D. (1995). Walking the Victorian Streets: Women, Representation, and the City. Ithaca, NY: Cornell University Press. 10.7591/9781501729232

[B50] Nuvolati G. (2006). Lo sguardo vagabondo. Il flâneur e la città da Baudelaire ai postmoderni. Bologna: il Mulino.

[B51] Nuvolati G. (2013). L'interpretazione dei luoghi. Flânerie come esperienza di vita. Firenze: Firenze University Press.

[B52] Nuvolati G. (2014). “The flaneur: A way of walking, exploring and interpreting the city,” in Walking in the European City. Quotidian Mobility and Urban Ethnography, eds T. Shortell and E. Brown (Burlington: Ashgate), 21–40.

[B53] Oomen B. (2019). Human rights cities: the politics of bringing human rights home to the local level, in Mobilising International Law for “Global Justice” (Cambridge: Cambridge University Press), 208. 10.1017/9781108586665.010

[B54] Oomen B. Baumgärtel M. (2018a). Frontier cities: the rise of local authorities as an opportunity for international human rights law. Eur. J. Int. Law 29, 607. 10.1093/ejil/chy021

[B55] Oomen B. Baumgärtel M. (2018b). Human Rights Cities. Available online at: https://www.philodroit.be

[B56] Pain R. (1991). Space, sexual violence and social control: integrating geographical and feminist analysis of women's fear of crime. Prog. Hum. Geogr. 15, 415–431. 10.1177/030913259101500403

[B57] Pain R. (2001). Gender, race, age and fear in the City. Urban Stud. 38, 899–913. 10.1080/00420980120046590

[B58] Pollock G. (1992). Modernity and the Spaces of Femininity. London: Routledge.

[B59] Predelli L. N. (2008). Religion, citizenship and participation: a case study of immigrant muslim women in Norwegian mosques. . Eur. J. Womens Stud. 15, 241–260. 10.1177/1350506808091506

[B60] Saccà F. (2018). Democracy, Power and Territories. Milano: Franco Angeli.

[B61] Schroeder K. (ed). (2021). Cities in International Law: the New Landscape of Global Governance. Virginia Journal of International Law, 61:364.

[B62] Secchi B. (2016). La città dei ricchi e la citt? dei poveri. Roma-Bari: Laterza.

[B63] Simmel G. (1890). Ü*ber Sociale Differenzierung*. Leipzig: Duncker and Humblot. 10.3790/978-3-428-57712-5

[B64] Singh S. (2005). Brandeis's happy incident revisited: U.S. cities as the new laboratories of international law. Geo. Wash. Int'l Rev. 37, 1–537.

[B65] Sisson R. Sanders R. (2021). Prospects for realizing international women's rights law through local governance: the case of cities for CEDAW. Hum. Rights Rev. 22, 303–325. 10.1007/s12142-021-00635-z PMC8417664 38624702

[B66] Soja E. W. (1996). Thirdspace: Journey to Los Angeles and Other Real-and-immaginated Places. Malden: Blackwell.

[B67] Soja E. W. (2000). Postmetropolis: Critical Studies of Cities and Regions. Hoboken, NJ: Wiley.

[B68] Swiney C. (2021). The urbanization of international law and international relations: the rising soft power of cities in global governance. Michigan J. Int. Law 41, 227. 10.36642/mjil.41.2.urbanization

[B69] Tastsoglou E. Dobrowolsky A. (eds) (2006). Women, Migration and Citizenship. Aldershot: Ashgate.

[B70] Tremblay M. (2000). Women, citizenship, and representation: an introduction. Int. Polit. Sci. Rev. 21, 339–343. 10.1177/0192512100214001

[B71] Valentine G. (1998). “Sticks and stones may break my bones”: a personal geography of harassment. Antipode 30, 305–332. 10.1111/1467-8330.00082

[B72] Wekerle G. (1984). A woman's place is in the city. Antipode 16, 11–19. 10.1111/j.1467-8330.1984.tb00069.x

[B73] Williams F. (2016). The Flâneuse. Appearing to be Invisible. Saarbücken: Lambert Academic Publishing.

[B74] Wilson E. (1992). The inivisible flâneur. New Left Rev. 191, 90–110.

[B75] Wolff J. (2006). The Invisible Flâneuse. Women and the Literature of Modernity. Style and Time: Essays on the Politics of Appearance. Evanston: Northwestern University Press.

[B76] Yuval-Davis N. (1997). Women, citizenship and difference. Fem. Rev. 57, 4–27. 10.1080/014177897339632

[B77] Zola E. (1883). Au Bonheur des Dames. Paris: Charpentier.

